# In Situ Damage Detection Method for Metallic Shear Plate Dampers Based on the Active Sensing Method and Machine Learning Algorithms

**DOI:** 10.3390/s26072203

**Published:** 2026-04-02

**Authors:** Yunfei Li, Feng Xiong, Hong Liu, Xiongfei Li, Huanlong Ding, Yi Liao, Yi Zeng

**Affiliations:** 1 College of Architecture and Environment, Sichuan University, Chengdu 610065, China; liyunfei_3@163.com (Y.L.); fxiong@scu.edu.cn (F.X.); 2 Chengdu Fourth Construction Engineering of CDCEG, Chengdu 610014, China; liuhong197010@163.com (H.L.); lixiongfei_12@163.com (X.L.); 3 Chengdu Architectural Design & Research Institute Co., Ltd., Chengdu 610041, China; dinghuanlong@cdadri.com (H.D.); liaoyi2021@cdadri.com (Y.L.); 4 Sichuan Provincial Key Laboratory of Seismic Safety and Resilience, Chengdu 610031, China

**Keywords:** metallic shear plate dampers, structural health monitoring, active sensing, stress wave analysis, wavelet packet transform, machine learning, k-nearest neighbor (KNN)

## Abstract

**Highlights:**

**What are the main findings?**

- An active sensing-based approach combined with wavelet packet energy features and k-nearest neighbor (KNN) enables accurate damage-state classification for metallic shear plate dampers (MSPDs).
- The proposed model achieves 98.9% validation accuracy using all data and a test accuracy of 92.6% for healthy–damaged state discrimination when applied to the validation specimen.

**What are the implications of the main findings?**

- The method provides a rapid, low-cost alternative to visual inspection and improves the detectability of concealed damage in post-earthquake damper assessment.
- The model demonstrates good generalization capability on structurally slightly different dampers, supporting the practical application of stress-wave-based structural health monitoring for dampers in real structures.

**Abstract:**

Metallic Shear Plate Dampers (MSPDs) are essential components in passive vibration control systems and require rapid post-earthquake inspection to assess damage and determine replacement needs. Traditional visual inspection methods suffer from low efficiency and limited ability to detect concealed damage. This study proposes a novel MSPD damage detection method based on active sensing and the k-nearest neighbor (KNN) algorithm, featuring high accuracy, efficiency, and low cost. Quasi-static tests were conducted to simulate various damage states. Sweep-frequency excitation was applied using a charge amplifier, and piezoelectric sensors were employed to generate and receive stress wave signals corresponding to different damage conditions. The acquired signals were processed using wavelet packet transform (WPT) and energy spectrum analysis to extract discriminative time–frequency features, which were used to train and validate the KNN model. Results show that the model achieved a validation accuracy of 98.9% using all valid data and 98.1% using a single excitation-sensing channel. When tested on an MSPD with a similar overall structure but lacking stiffeners, the model achieved an accuracy of 92.6% in distinguishing between healthy and damaged states. This indicates that the proposed method has good robustness and practical potential for MSPDs with similar damage evolution and failure modes despite certain structural variations.

## 1. Introduction

MSPDs are typical passive energy dissipation devices, commonly used for energy dissipation in the energy-dissipating coupling beams of shear wall structures [1,2] or interlayer braces in frame structures [3,4,5]. The damper web is composed of low-yield-point steel, which is characterized by a high deformation capacity, excellent hysteretic performance, strong energy dissipation capability, and ease of replacement [6].

Under seismic loading, inelastic deformations are concentrated in the seismic dampers, and the damage in the main structure can be drastically reduced or eliminated [7,8]. To maintain the structure’s seismic efficacy, it is essential to promptly evaluate the condition of the dampers after an earthquake to determine whether replacement is warranted. Manual inspection is the conventional method employed for damage detection in MSPDs due to its simplicity and practicality. However, this method suffers from several limitations: (i) it exhibits low efficiency, especially since some dampers are located in concealed or interior areas of the structure; (ii) its damage identification accuracy is limited, making it difficult to identify the damage cumulative state [9]. Thus, a high-accuracy detection method that does not rely on visual inspection needs to be proposed.

Structural Health Monitoring (SHM) technology provides an effective framework for damage identification in engineering structures and has been extensively studied in the broader field of damage detection. By installing displacement transducers [10], accelerometers [11], and strain gauges [12] on the MSPDs, it is possible to obtain changes in the mechanical parameters of the component, such as stiffness and energy dissipation capacity, thus enabling efficient and accurate damage identification of the dampers [13,14,15]. However, these SHM-based approaches are typically designed for global structural assessment rather than component-level detection [16,17,18]. Their application to MSPDs is limited by the need for continuous, long-term monitoring, dense sensor deployment, and complex data acquisition and transmission systems, which result in high maintenance and operational costs [9].

To address the limitations of the traditional SHM methods, active sensing, as an advanced nondestructive testing technique, has been increasingly applied to damage detection in various engineering materials and structural components. By using piezoelectric materials as sensors, this technique enables post-earthquake inspection of devices and provides damage-related information for evaluating damper conditions [19,20]. Unlike traditional SHM approaches that rely on continuous long-term monitoring, active sensing allows targeted inspection at specific stages, thereby reducing operational and maintenance costs. Due to its stable signal, cost-effectiveness, and ease of implementation, this method has been widely applied in fields such as composite material damage detection [21] and damage detection in concrete-filled steel tube structures [22,23]. A. Gallego et al. used the active sensing method to detect metallic bending plate dampers (MBBD) with different levels of artificial damage, and established a mapping relationship between the damage index (Area Index Damage) calculated from the detection signals and the damper’s damage states, effectively reflecting the condition of the damper [24]. In addition, machine learning (ML) algorithms have been widely applied in structural damage detection [25,26,27], effectively simplifying the identification process and reducing the cost of damage detection. Yi Zeng et al. proposed a linear discriminant analysis (LDA) method based on wavelet packet energy spectrum for detecting rubber fracture damage [28]. Chubing Deng et al. introduced a high-precision void damage detection model based on the BO-XGBoost algorithm [29]. Mojtaba Salkhordeh et al. utilized features extracted from structural acceleration responses and classification learners to assess the health condition of braced-frame buildings [30]. They also proposed a rapid damage detection framework based on machine learning to detect the extent of damage to both primary and secondary components of reinforced concrete bridges subjected to seismic loading [31]. Among various Machine Learning methods, the KNN algorithm has attracted attention in structural health monitoring because of its simplicity, ease of implementation, and good performance on small- and medium-sized datasets. Wang et al. developed a KNN-based damage stage evaluation method for bridges using acoustic emission parameters [32], and Sousa et al. proposed KNN-based approaches for damage detection and quantification in adhesive joints using electromechanical impedance measurements [33]. By combining the active sensing method with machine learning algorithms, the proposed framework has the potential to improve the accuracy and efficiency of MSPD damage detection, thereby providing a more efficient and reliable solution for structural health monitoring.

Based on this, this paper proposes a new damage detection method for MSPDs. First, the MSPDs are subjected to quasi-static loading to create different damage states, while detection signals are obtained under various damage states using the active sensing method, with sensors placed on the end plates where deformation is minimal. Then, the signals are processed using a wavelet packet transform to extract wavelet packet energy spectrum features, and a damage prediction model for the damper is established based on machine learning algorithms. Finally, the damper’s damage state is predicted using the damage prediction model. Based on this mapping relationship, the proposed method can achieve rapid and reliable identification of the damage states of MSPDs of the same type, even in the presence of minor structural differences.

## 2. Method

**Figure 1:**
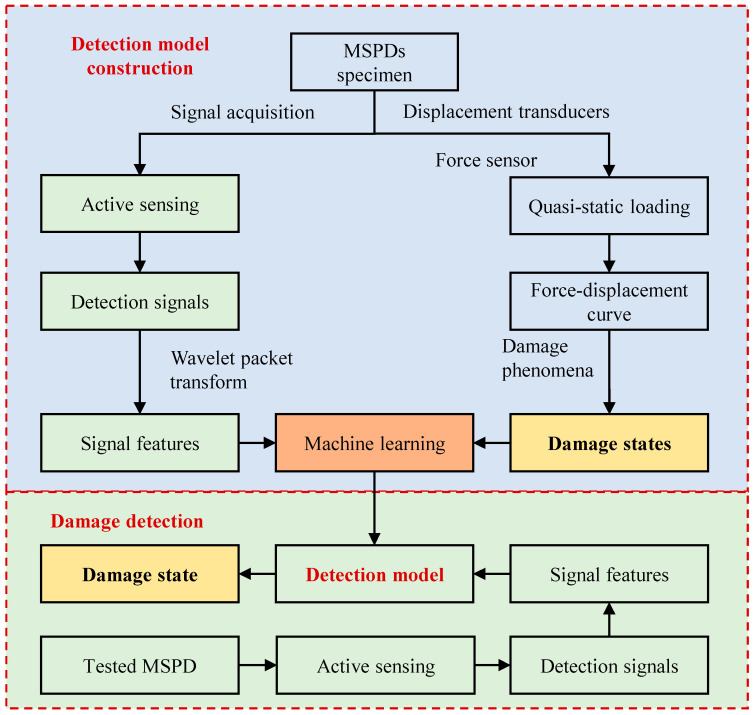
Damage detection method of MSPDs.

The MSPD’s damage detection method based on active sensing and machine learning algorithms consists of two main parts: detection model construction and model application, as shown in Figure 1.

For a specific type of MSPD, the damage states and corresponding damage signal features are obtained through quasi-static tests and active sensing methods. During the quasi-static cyclic loading tests, the typical damage evolution process of this type of damper is reproduced. Based on the obtained hysteretic curves and skeleton curves, the stiffness variation and degradation of the damper are analyzed, and the damage states are classified according to the stiffness changes together with the observed damage phenomena. At the same time, active sensing is used to collect the stress wave signals corresponding to different damage states. However, the raw signals contain a large amount of data, and directly using them for modeling and identification would result in excessively high input dimensionality, which may further lead to an increased model size, longer training time, and reduced computational efficiency. Therefore, the maximum amplitudes of the time-domain and frequency-domain signals, together with the wavelet packet energy spectrum features extracted by the wavelet packet transform, are taken as the model inputs, while the damage states determined from the quasi-static tests are used as the output labels, in order to establish a damage detection model for this type of MSPDs. In the model application stage, active sensing is used to obtain the damage signal features of the target MSPD, and these features are then input into the trained damage detection model to predict its damage state.

**Figure 2:**
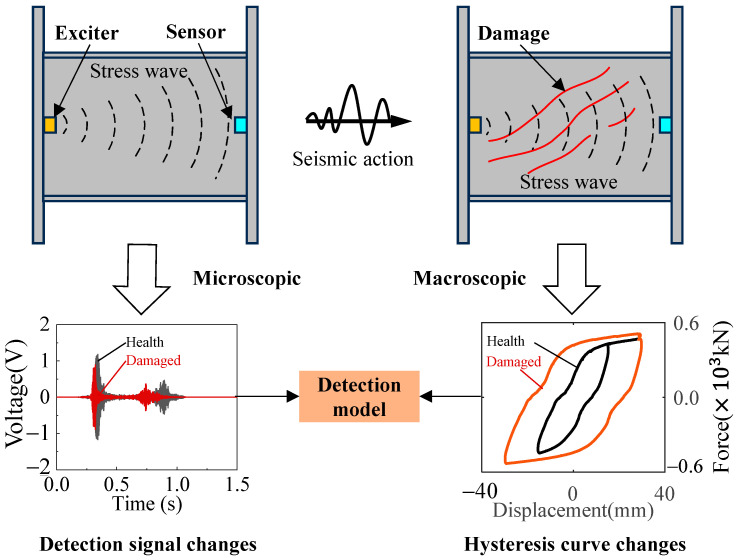
The detection principle of the active sensing method.

The principle of the active sensing method used for damage detection in MSPDs is illustrated in Figure 2. Piezoelectric sensors (exciters and sensors) are attached to the surface in the end plates, forming an excitation-sensing channel with the structure through the direct and inverse piezoelectric effects of the piezoelectric ceramics. Under seismic loading, the web of the MSPD gradually transitions from the elastic stage to the plastic stage, and microscopic damage features such as plastic accumulation, micro-cracks, and material discontinuities are formed in local regions. At the microscopic level, the initiation of micro-cracks and local material discontinuities in the web alters the propagation characteristics of high-frequency stress waves, thereby causing variations in the waveform, amplitude, and frequency-band energy distribution of the detected signals. At the macroscopic level, with the initiation and propagation of micro-cracks, the mechanical performance of the damper gradually deteriorates, mainly manifested by stiffness reduction, evolution of energy dissipation characteristics, and changes in hysteretic response features. Therefore, variations in the active sensing signals can, to a certain extent, reflect the degradation process of the damper’s mechanical performance, thus providing a basis for damage-state identification and the establishment of the damage detection model. In the in situ detection of dampers, the damage data used for model development can be obtained during the mechanical performance testing of the dampers. Since dampers are typically subjected to sampling inspection before delivery, which involves mechanical performance testing, the damage dataset can be established simultaneously during this process, enabling integrated testing and data collection.

## 3. Experiment Setup

### 3.1. Specimens

**Figure 3:**
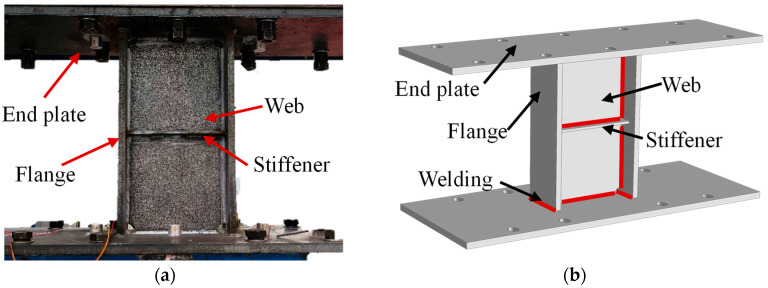
The structure of the MSPDs. (**a**) Specimen of MSPDs; (**b**) Schematic diagram of MSPDs.

As shown in Figure 3, the MSPD is composed of four components: the end plates, flanges, web, and stiffener, all of which are connected by welding. Energy dissipation in MSPDs is primarily achieved through shear plastic deformation of the web. In the specimen, the web is fabricated from LY225 low-yield-point steel, which is characterized by relatively low yield strength, good ductility, and stable plastic deformation capacity, making it suitable for the energy-dissipating part of the damper. In contrast, the end plates, flanges, and stiffeners are made of Q355 structural steel, which has higher strength and is used to ensure that these components remain essentially elastic while the web undergoes inelastic deformation.

The main mechanical properties of LY225 and Q355, including yield strength, ultimate tensile strength, and elastic modulus, are summarized in Table 1.

**Figure 4:**
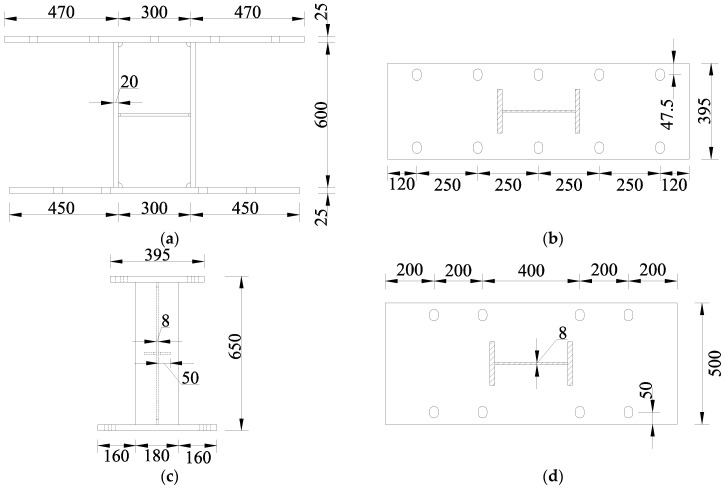
Dimensions of each component of the specimen. Unit: mm. (**a**) Front view; (**b**) Upper end plate; (**c**) Side view; (**d**) Lower end plate.

Figure 4 shows the detailed dimensions of each component of the specimen. The dimensions of the web are 300 mm × 600 mm, with a thickness of 8 mm. To resist bending moments, the flange is designed with dimensions of 180 mm × 600 mm and a thickness of 20 mm. The end plates were modified to accommodate the loading plates of the quasi-static testing apparatus; the upper and lower end plates have dimensions of 395 mm × 1240 mm and 500 mm × 1200 mm, respectively. To ensure the end plates remain in the elastic state during loading, their thickness was set to 25 mm. In order to prevent premature buckling and failure of the web, a stiffener was added at the center of the web, with dimensions of 50 mm × 300 mm and a thickness of 10 mm. Additionally, grooves were introduced at the welded joints between the web and the stiffener to reduce stress concentration during the loading process.

**Figure 5:**
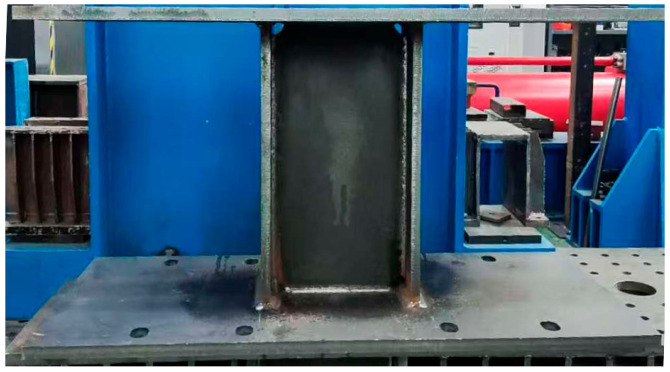
Validation MSPD specimen.

In addition to the primary specimen, a validation MSPD with identical dimensions but without stiffeners was introduced (Figure 5) to assess the applicability of the proposed damage detection model for MSPDs with similar damage evolution and failure modes.

### 3.2. Experiment Scheme

#### 3.2.1. Testing Equipment and Loading Protocol

**Figure 6:**
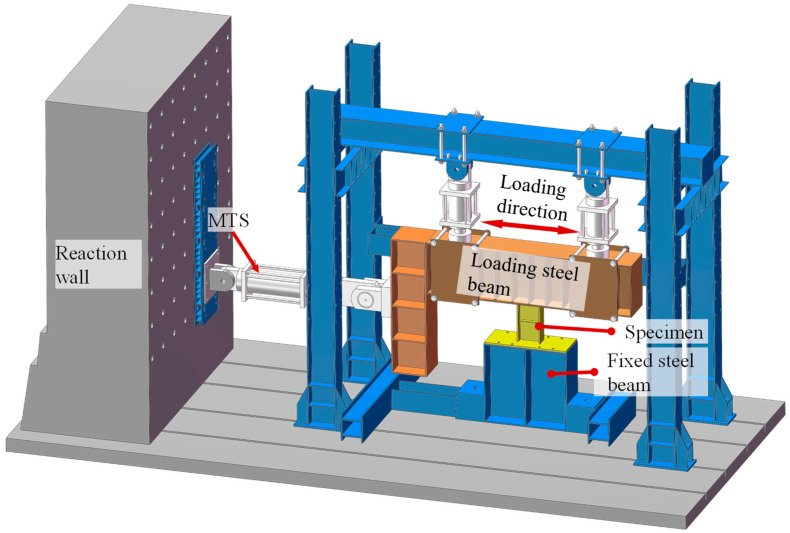
Experimental Setup.

The JAW-2000J multi-channel combination loading frame is used in this experience, which has both force and displacement control modes. The experimental setup is shown in Figure 6 and primarily consists of a fixed steel beam, a loading steel beam, and the MTS servo system. The lower end plate of the metallic damper is fixed to the support system to simulate realistic boundary conditions, while the upper end plate is bolted to the loading steel beam. When the loading beam moves along the specified direction, the damper mainly experiences shear forces, thereby simulating its actual working condition under seismic action. The main specifications of the loading frame are as follows: vertical compression capacity 2000 kN, vertical tensile capacity 1400 kN, vertical stroke 300 mm, vertical pushing capacity 1500 kN, horizontal tensile capacity 1000 kN, horizontal stroke ±250 mm, and maximum test height 4125 mm. These capabilities are sufficient to perform the quasi-static cyclic loading required for the damper. During the test, controlled cyclic loading sequentially induces different damage states of the damper, from elastic deformation to yielding and ultimately to failure, while providing reliable experimental data for the piezoelectric sensors to collect signals for subsequent wavelet packet feature extraction and machine learning model training.

**Figure 7:**
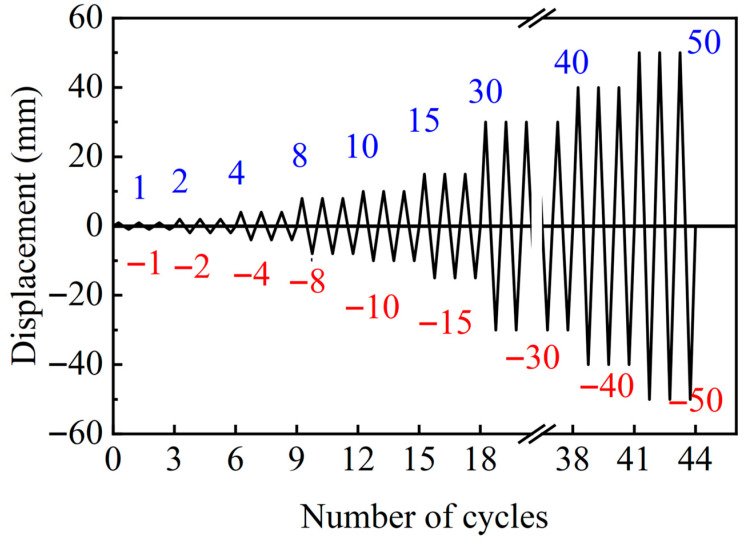
Loading Scheme.

In accordance with the Chinese code of the “Specification for seismic test of buildings”, the loading protocol for the quasi-static test should adopt a dual control approach based on both load and deformation. Since the web of the specimen is fabricated from the low-yield steel LY225 and yields at relatively small displacements, deformation control can be directly implemented. The deformation value is set as the maximum displacement of the specimen at yield, and loading is controlled by using multiples of this displacement as the range, with each load level being applied three times. The horizontal actuator moves at a displacement rate of 1 mm/min for each cycle. The vertical actuator is used only to support and position the loading beam and does not directly apply the load. When the displacement loading reaches 30 mm, the specimen is subjected to fatigue loading to observe changes in its energy dissipation capacity. After the energy dissipation capacity declines, the loading displacement is further increased to induce failure, thereby allowing investigation of the piezoelectric signal variations post-damage. Consequently, the mechanical performance tests are conducted using cyclic loading, with the loading levels and corresponding designations detailed in Figure 7.

#### 3.2.2. Experimental Measurement

**Figure 8:**
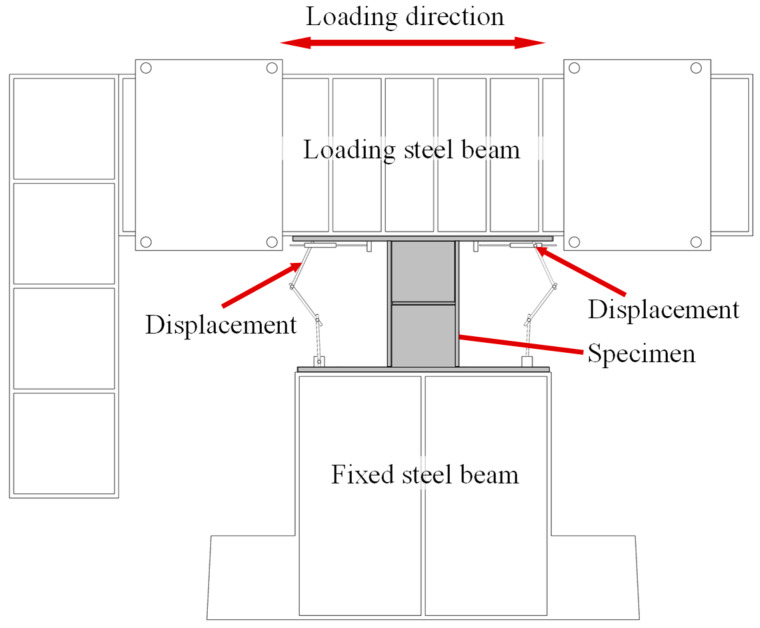
The place of the displacement transducers.

**Figure 9:**
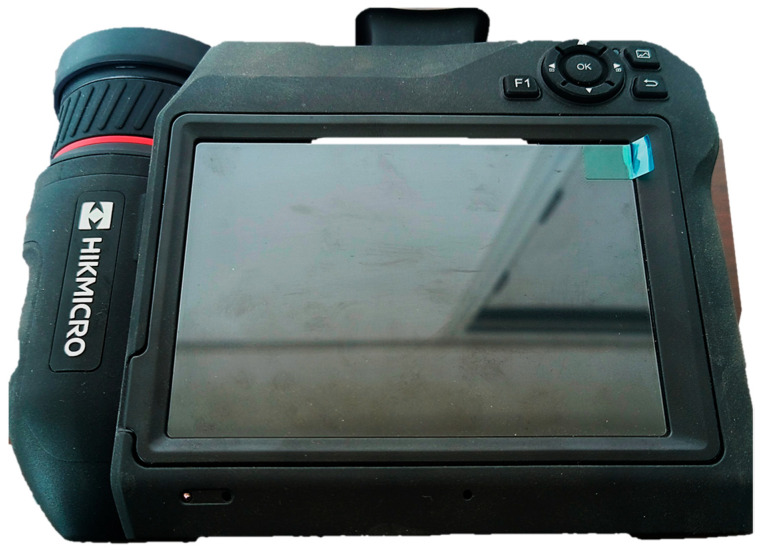
Handheld infrared thermometer.

As shown in Figure 8, two displacement transducers are installed at both ends of the damper to continuously record the relative displacement between the upper and lower end plates of the damper (from the top of the lower end plate to the bottom of the upper end plate). The bearing capacity is measured using the force measurement equipment of the MTS system. At the same time, a thermal imager (see Figure 9) was used to record the temperature changes in the web under different damage states. The thermal imager employed in this study is a Hikmicro HM-TP9GL-CQ964 (Hikmicro, Hangzhou, China), featuring a resolution of 640 × 480 pixels, a NETD of <0.03 °C, and a frame rate of 50 Hz.

**Figure 10:**
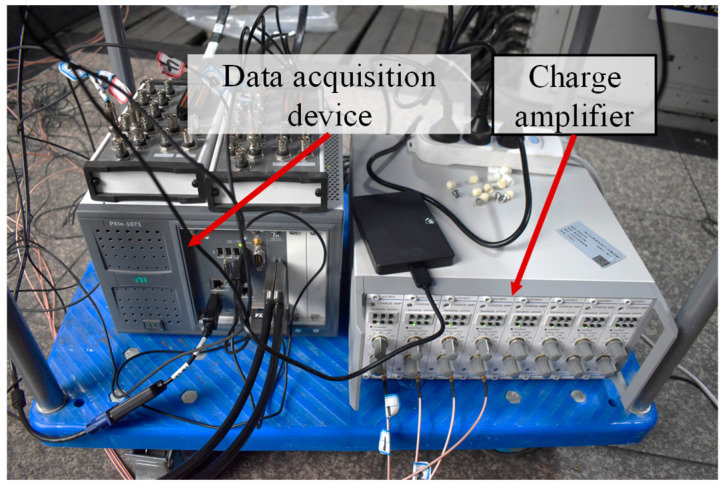
Active Sensing Experiment.

The active sensing experiment was conducted using the test equipment shown in Figure 10, which includes a data acquisition device and a charge amplifier. The model of the data acquisition device is NI PXIe-1071 (National Instruments Corporation, Austin, TX, USA), which has two modules: one for generating excitation signals and the other for collecting vibration signals. The charge amplifier YE5853 (LianNeng Electronics, Beijing, China) converts sensors charge into voltage signals and amplifies them.

**Figure 11:**
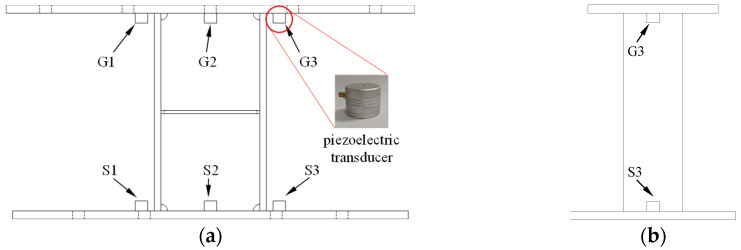
Positions of the Piezoelectric Transducers. (**a**) Front view; (**b**) Side view.

Three piezoelectric transducers are arranged on both the upper and lower end plates of the damper, with their positions shown in Figure 11. The transducers on both sides of the end plates are positioned 20 mm away from the flange plates, aligned with the center of the flange plates. The transducer at the center of the end plate is positioned 20 mm from the web. The transducers on the upper end plate, labeled as G1–G3, function as exciters and are responsible for receiving electrical signals and converting them into high-frequency vibration signals. The transducers on the lower end plate, labeled as S1–S3, serve as sensors, receiving the high-frequency vibration signals and converting them back into electrical signals. The piezoelectric transducers were made of PZT material, with a relative dielectric constant of approximately 1700, a piezoelectric constant d_33 ranging from 190 to 450 pC/N, and a resonant frequency of 58 kHz.

**Figure 12:**
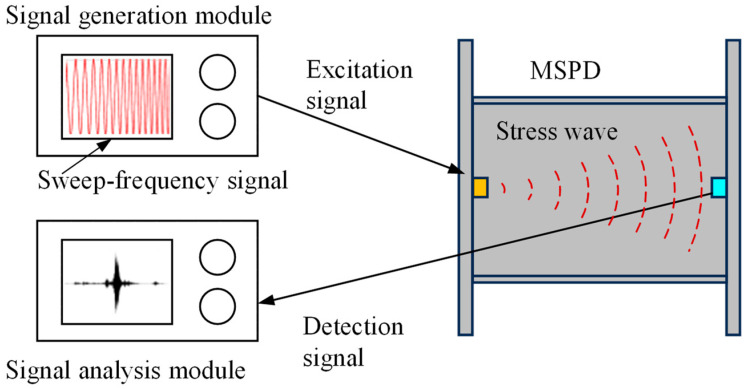
The connection configuration of the active sensing detection system.

The connection configuration of the active sensing detection system is shown in Figure 12. The signal generation module generates the excitation signal, which is converted into a stress wave propagating through the damper by the exciter. The signal is then received by the sensor and transmitted to the signal processing module for analysis.

During each active sensing test, six types of excitation signals are sequentially applied to the transducers on the upper end plate and received by those on the lower end plate. The excitation signals are sweep-frequency signals generated by the data acquisition system, with an excitation duration of 1 s and an amplitude of ±10 V. The specific frequency ranges of each signal are listed in Table 2. The signals are sampled at 1 MHz with a total acquisition time of 1.5 s, starting 0.2 s before the excitation to ensure the complete recording of the vibration signals. Additionally, when the specimen exhibits significant damage, the loading is paused, and a wave propagation measurement is conducted.

## 4. Experimental Results

### 4.1. Damage and Failure Modes

Damage Evolution and Classification of Damage States

**Figure 13:**
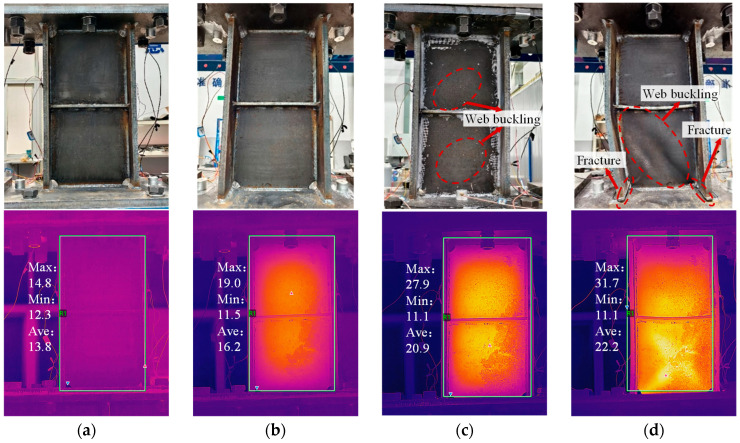
Damage states and corresponding infrared thermography images. (**a**) Healthy; (**b**) Damage progression; (**c**) Web buckling; (**d**) Failure.

During the quasi-static cyclic loading, the damage evolution of the primary specimen was first monitored through visual inspection and infrared thermography. At small displacements (0–4 mm), no visible deformation was observed in the web or end plates, and the maximum temperature of the web remained close to the ambient temperature of ~14.8 °C, as shown in Figure 13a, indicating that the web had not yet entered the plastic stage and that energy dissipation was limited. When the displacement increased to 15 mm, as shown in Figure 13b, uniform heating appeared at the center of the web, with a peak temperature reaching 19 °C, and slight coating peeling was observed. However, no apparent structural damage was visible, indicating that the web’s energy dissipation had increased, it had entered the plastic stage, and micro-cracks had begun to form. During the subsequent 30 mm displacement cycles, as shown in Figure 13c, the web exhibited noticeable buckling, the energy dissipation continued to increase, and the maximum temperature rose to 27.9 °C, suggesting that the micro-cracks had further developed and the energy dissipation capacity progressively increased. At the 38th cycle (displacement 30 mm), noticeable cracks appeared at the welds between the flange and end plate, the energy dissipation decreased, and the temperature rose to 31.7 °C, as shown in Figure 13d, indicating that the damper had started to fail. As the displacement increased to 40 mm, the web buckling intensified, and the weld cracks continued to propagate. When the displacement reached 50 mm, during the second cycle of cyclic loading, the load suddenly dropped, severe buckling occurred in the lower web and flange, and the welds completely fractured, marking the complete failure of the damper.

**Figure 14:**
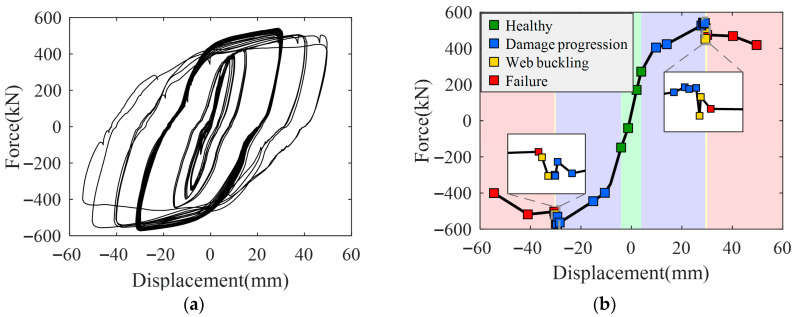
The hysteresis curve and skeleton curve of the primary specimen. (**a**) The hysteresis curve; (**b**) The skeleton curve.

The hysteresis and skeleton curves of the primary specimen are shown in Figure 14. When the displacement was in the range of 0–4 mm, the hysteresis loops were well-closed with a small loop area, indicating that the damper remained in the elastic stage. The peak load in this stage was approximately 265 kN, and the tangent stiffness derived from the skeleton curve was about 66.3 kN/mm. Energy dissipation was minimal, and the structure remained intact. When the displacement increased to 4–30 mm, the hysteresis loops gradually opened, and the loop area increased significantly, indicating that the damper had entered the plastic stage and was dissipating energy. The peak load reached approximately 543 kN, while the tangent stiffness decreased to around 20.9 kN/mm. Micro-cracks began to develop in the web, and energy dissipation progressively increased. During the fatigue loading stage (31st–36th cycles, displacement 30 mm), the peak load gradually decreased to approximately 466 kN, indicating that the damper’s stiffness continued to degrade and its energy dissipation reached a relatively stable state, although it had not yet fully failed. Finally, when the displacement reached 50 mm, the peak load dropped to approximately 400 kN, the hysteresis loops opened significantly, severe web buckling occurred, and cracks appeared and fully propagated in the welds, indicating complete failure of the damper.

Based on the variations in the damper’s mechanical performance during cyclic loading, as well as the energy dissipation and stiffness degradation reflected by the hysteresis and skeleton curves, and combined with the observed deformation and failure phenomena (such as micro-cracks in the web, buckling, and crack propagation in welds), the behavior of the MSPD can be classified into four damage states: (i) D0: Healthy state: the damper is in the elastic stage, the hysteresis loops are well-closed, the stiffness of the skeleton curve remains nearly constant, and energy dissipation is minimal; (ii) D1: Damage progression state: the damper enters the plastic stage, the hysteresis loops gradually open, the stiffness of the skeleton curve decreases, micro-cracks appear in the web, and energy dissipation increases significantly; (iii) D2: Web buckling state: under large displacement cycles, the web undergoes noticeable buckling deformation, the hysteresis loops open further, and energy dissipation reaches a relatively stable level; and (iv) D3: Failure state: the damper’s peak load drops significantly, the hysteresis loops open markedly, severe web buckling occurs, weld cracks propagate completely through the welds, and the damper fails. The corresponding loading displacements and cycle numbers for each damage state are summarized in Table 3.

**Figure 15:**
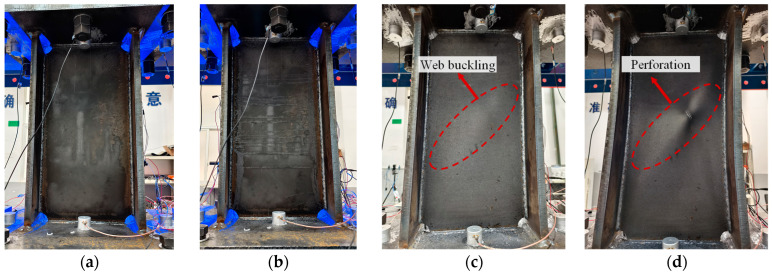
Damage states of the validation specimen. (**a**) Healthy; (**b**) Damage progression; (**c**) Web buckling; (**d**) Failure.

The damage evolution of the validation specimen is shown in Figure 15. At small displacements (0–4 mm), no visible deformation was observed in the web or end plates, and the web had not yet entered the plastic stage. As the displacement gradually increased, slight peeling of the web surface coating was observed, but the overall structure remained intact, indicating that energy dissipation had begun to increase without significant damage. When the displacement reached 10 mm, noticeable buckling appeared on the web surface, micro-cracks began to develop, and the energy dissipation capacity further increased. During the fatigue loading stage (displacement 30 mm), at the 11th cycle, perforation of the web occurred, marking severe damage and complete failure of the damper.

**Figure 16:**
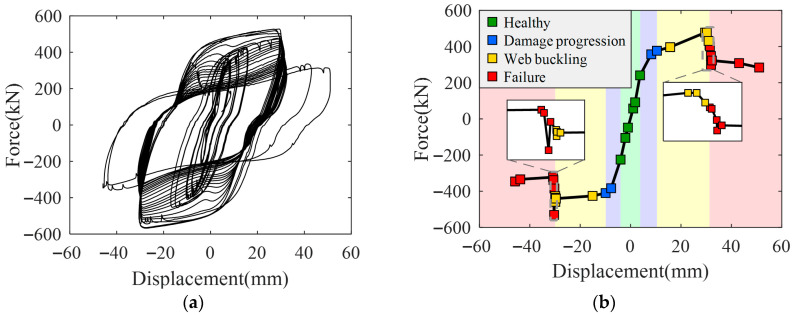
The hysteresis curve and skeleton curve of the validation specimen. (**a**) The hysteresis curve; (**b**) The skeleton curve.

The hysteresis and skeleton curves of the validation specimen are shown in Figure 16. When the displacement was in the range of 0–4 mm, the damper remained in the elastic stage, with a peak load of approximately 255 kN and a tangent stiffness of about 62.3 kN/mm. As the displacement increased to 10 mm, the peak load rose to approximately 377 kN, while the tangent stiffness decreased to around 20.4 kN/mm, and noticeable buckling appeared in the web. When the displacement further increased to 30 mm, the peak load remained approximately 477 kN, the tangent stiffness stayed around 5 kN/mm, and web buckling became more pronounced. During the fatigue loading stage in the 11th week (displacement 30 mm), the peak load gradually decreased to approximately 408 kN, local perforation appeared in the web, and the damper had reached a failed state. Unlike the primary specimen, due to the lack of rib constraints, the web of the validation damper buckled at an earlier stage, leading to increased local stress concentrations and accelerated initiation and propagation of micro-cracks. Consequently, the overall damage evolution progressed more rapidly, the energy dissipation reached a relatively stable level earlier, and both the load-bearing capacity and fatigue life of the damper were significantly reduced.

Based on the variations in mechanical performance and observed damage phenomena, the loading displacement ranges and cycle numbers corresponding to each damage state of the validation specimen are summarized in Table 4.

### 4.2. Detection Signal Analysis

For the primary specimen, a total of 18 active sensing tests were conducted throughout the experiment. In each test, the exciter applied the same type of excitation signal three times consecutively. The first test was performed at 0 mm displacement, followed by one test after every three loading cycles at each displacement level. Additionally, an extra test was conducted after the completion of loading at the 30 mm displacement level. For the 50 mm displacement level, three tests were carried out: after loading was stopped, after unloading to zero displacement, and after unloading to zero force. In total, the numbers of active sensing tests corresponding to the four damage states were 4, 7, 2, and 5, respectively. For each signal type under the same excitation-sensing channel, 54 valid data samples were obtained. Due to the malfunction of sensor S3, only six excitation-sensing channel combinations were available, and a total of 1944 valid data samples were collected under six types of excitation signals.

For the validation specimen, 54 data samples were used for testing, which were acquired from Signal-0 on the G1–S1 channel.

#### 4.2.1. The Effect of Damage States on the Detection Signals

**Figure 17:**
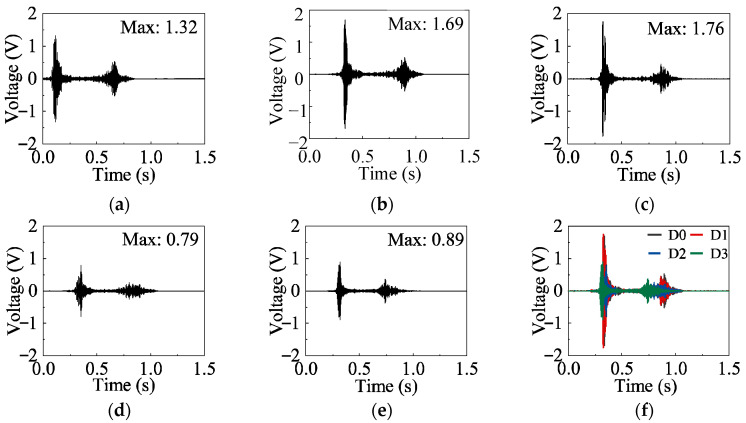
Time-domain signals under different damage states. Excitation: Signal-0, G1–S1. (**a**) Healthy (0 mm); (**b**) Healthy (2 mm); (**c**) Damage progression (30 mm); (**d**) Web buckling (30 mm); (**e**) Failure (50 mm); (**f**) Different damage states.

When the exciter is G1, the sensor is S1, and the excitation signal is Signal-0, the time-domain plots corresponding to the maximum amplitude in each damage state are shown in Figure 17. The waveforms of the detection signals in the time-domain are generally similar across different damage states, but the maximum amplitude decreases progressively with increasing damage severity.

In the healthy state, the maximum amplitude of the time-domain signal ranges from 1.18 V to 1.68 V, with the peak amplitude corresponding to a displacement of 2 mm. In the damage progression state, the maximum amplitude ranges from 1.36 V to 1.76 V, with the peak amplitude corresponding to a displacement of 30 mm. In the web buckling state, the maximum amplitude falls between 0.65 V and 0.79 V. In the failure state, the maximum amplitude ranges from 0.45 V to 0.89 V, with the highest value observed at a displacement of 50 mm when the load is unloaded to zero.

The time-domain signals can, as a whole, reflect the influence of damage evolution on the specimen response; that is, the amplitude of the detected signals generally shows a decreasing trend with increasing damage severity. However, from the perspective of damage-state identification, relying solely on the maximum amplitude still has limitations: in the early stage of damage development, the maximum amplitudes of the healthy state and the damage progression state are relatively close, whereas in the later severe damage stage, the amplitude ranges of the web buckling state and the failure state overlap. Therefore, although the time-domain signals can provide preliminary information on damage evolution, they are not sufficient on their own to accurately distinguish different damage states.

**Figure 18:**
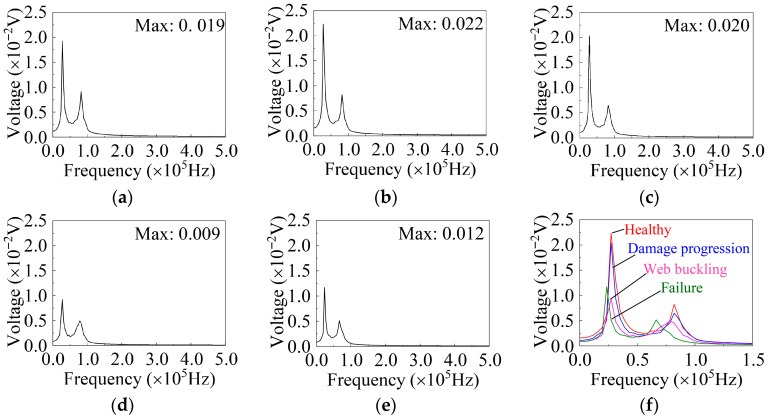
Frequency-domain signals under different damage states. Excitation: Signal-0, G1–S1. (**a**) Healthy (0 mm); (**b**) Healthy (4 mm); (**c**) Damage progression; (**d**) Web buckling; (**e**) Failure; (**f**) Different damage states.

In the frequency-domain signals, as shown in Figure 18, all damage states exhibit two distinct peak points, with the characteristic frequency corresponding to the maximum amplitude approximately at 27,343.75 Hz. In the healthy state, the amplitude ranges from 0.017 V to 0.022 V, with the highest value observed at a displacement of 4 mm. In the damage progression state, the amplitude range remains relatively stable, between 0.016 V and 0.020 V, with the maximum value occurring at a displacement of 30 mm. After web buckling and specimen failure, the maximum amplitude in the frequency domain significantly decreases, with the highest value observed when the load is unloaded to zero at 50 mm displacement. Similar to the time-domain signals, the maximum amplitude of the frequency-domain signals is also insufficient for robust damage-state identification. Specifically, the amplitude ranges of the healthy state and the damage progression state overlap, while the web buckling state and the failure state may also exhibit overlapping amplitudes or non-monotonic relationships in some tests. Therefore, relying solely on the maximum frequency-domain amplitude as a single indicator is still insufficient to reliably distinguish all damage states. The maximum amplitude of different damage states in each test is shown in Figure 18f.

This indicates that, in the early stage of damage development, the influence of micro-cracks and local discontinuities on the signals is mainly reflected in local variations in time-domain or frequency-domain features, while the overall amplitude change remains insignificant. Therefore, it is difficult to achieve reliable identification using only a single amplitude-based indicator. As the damage severity further increases, the differences in the detected signals generally become more pronounced; however, in the severe damage stage, the signal features corresponding to web buckling and complete fracture still exhibit some overlap. Therefore, more discriminative time–frequency features need to be further extracted.

**Figure 19:**
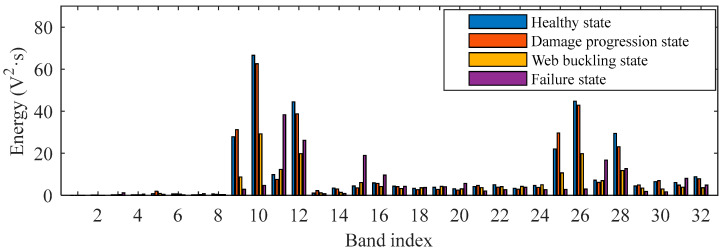
The wavelet packet energy spectrum under different damage states (from 1 to 32). Excitation: Signal-0, G1–S1.

Figure 19 shows the comparison of wavelet packet energy spectra from 1 to 32 under different damage states for the G1–S1 sensor channel with Signal-0 excitation (with 8 decomposition levels and the “db5” wavelet packet base). In the healthy state and damage progression state, the maximum amplitude ranges from 50 V^2^·s to 80 V^2^·s, with the highest value observed at a displacement of 4 mm. In the web buckling and failure states, the maximum amplitude significantly decreases to below 50 V^2^·s, with the highest value at this stage observed during unloading to zero force after 50 mm displacement loading. Compared with the time-domain and frequency-domain information, the wavelet packet energy spectrum exhibits more pronounced differences in shape under different damage states, although it is still difficult to directly determine the damage condition based solely on amplitude variations. Moreover, while time-domain and frequency-domain signals can also be used in machine learning algorithms, directly using the full signals would introduce high-dimensional inputs. In contrast, the wavelet packet energy spectrum provides a more compact and damage-sensitive feature representation, making it more suitable for efficient damage-level identification in the present study.

#### 4.2.2. The Effect of Excitation Signal Types on Detection Signals

**Figure 20:**
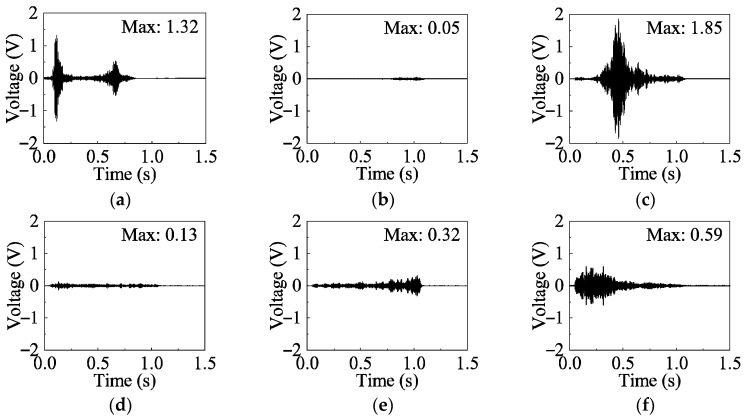
Time-domain signal in different signal types (G1–S1). (**a**) Signal-0; (**b**) Signal-1; (**c**) Signal-2; (**d**) Signal-3; (**e**) Signal-4; (**f**) Signal-5.

As shown in Figure 20, the effect of different types of excitation signals on the time-domain detection signals of the G1–S1 channel in the healthy state (0 mm) is illustrated. The detection signals generated under different sweep excitation signals exhibit significant differences in waveform and amplitude. Among them, when the excitation signal is Signal-2, its peak amplitude not only exceeds that of Signal-0 across the entire frequency band but also presents a distinct single-peak characteristic. In contrast, the waveforms of Signal-3, Signal-4, and Signal-5 show smaller amplitude variations and less pronounced time-domain features, while Signal-1 performs the worst overall, with the smallest amplitude variation and the least distinguishable time-domain signal characteristics.

**Figure 21:**
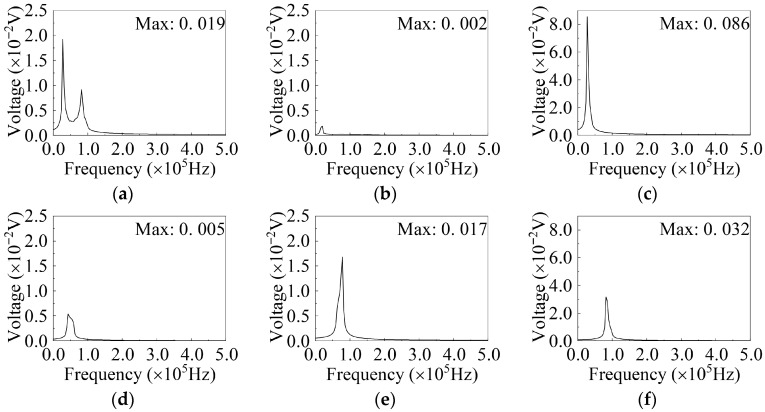
Frequency-domain signal in different signal types (G1–S1). (**a**) Signal-0; (**b**) Signal-1; (**c**) Signal-2; (**d**) Signal-3; (**e**) Signal-4; (**f**) Signal-5.

In the frequency domain (Figure 21), Signal-0 exhibits two distinct peak points at 27,343.75 Hz and 85,937.5 Hz, which correspond to the peak frequencies observed in Signal-2 and Signal-5, respectively.

This observation highlights the importance of selecting an appropriate sweep excitation frequency band for accurate damage detection of metallic shear plate dampers. Specifically, when the excitation frequency band encompasses the resonant frequencies of the specimen, the resulting signals exhibit enhanced amplitudes and more pronounced feature variations across different damage states, thereby improving the sensitivity and reliability of damage identification. The specimen can be analyzed using detection signals from the frequency bands of Signal-0, Signal-2, and Signal-5, with Signal-0 being primarily selected for analysis in this study.

## 5. Metallic Shear Plate Dampers Damage Identification

### 5.1. Prediction Model

As shown in Section 4.2.1, MSPDs exhibit significantly different signal characteristics under different damage states, indicating a certain degree of separability among different damage categories. Therefore, the identification of damage states can be regarded as a supervised multi-class classification problem. As a classical and effective pattern recognition method, the k-nearest neighbor (KNN) algorithm is suitable for distinguishing different damage states based on the extracted signal features.

In this study, multiple KNN-based damage classification models were developed to compare their damage identification performance under different input features and to further evaluate their generalization ability across different specimens. In addition, several other commonly used machine learning algorithms were selected as benchmark models to further analyze the performance differences and applicability of different algorithms in the damage detection task for metallic shear plate dampers.

#### Evaluation Indicators

After model training is completed, the Accuracy, Precision, and Recall on the training set, validation set, and test set are used as performance evaluation metrics. The formulas are as follows:

(1) $$Accuracy=\frac{N_{c}}{T},$$

where $N_{c}$ presents the count of samples correctly classified by the model; T presents the total number of samples.

(2) $$Precision_{i}=\frac{TP_{i}}{TP_{i}+FP_{i}},$$

where $TP_{i}$ is the number of true positives for class i and $FP_{i}$ is the number of false positives for class i.

(3) $$Recall_{i}=\frac{TP_{i}}{TP_{i}+FN_{i}},$$

where $FN_{i}$ is the number of false negatives for class i.

### 5.2. Damage Detection Results

#### 5.2.1. Comparison of Different Input Features

As indicated in Section 4.2, the dataset used in this experiment consists of a total of 1944 data records. The numbers of data records corresponding to the four damage states are 432, 756, 216, and 540, respectively. These data records were classified using the KNN algorithm, and 10-fold cross-validation was employed for model validation. Specifically, the dataset was divided into 10 subsets, each containing 194 or 195 data records. In each iteration, 9 subsets were selected as the training set and the remaining subset was used as the validation set. This process was repeated 10 times to ensure a comprehensive evaluation.

For the time-domain and frequency-domain cases, only the maximum amplitude of the corresponding signal was used as the input feature, so the input size of the KNN model was 1. These single-feature inputs were used to establish the mapping relationship between the extracted signal amplitudes and the damage states of the MSPDs. As shown in Table 5, the KNN models using time-domain and frequency-domain features achieved accuracies of 53.0% and 66.5%, respectively. These results indicate that KNN models trained using only the maximum amplitude in either the time or frequency domain as features are insufficient for effectively distinguishing the damage states of the dampers.

**Figure 22:**
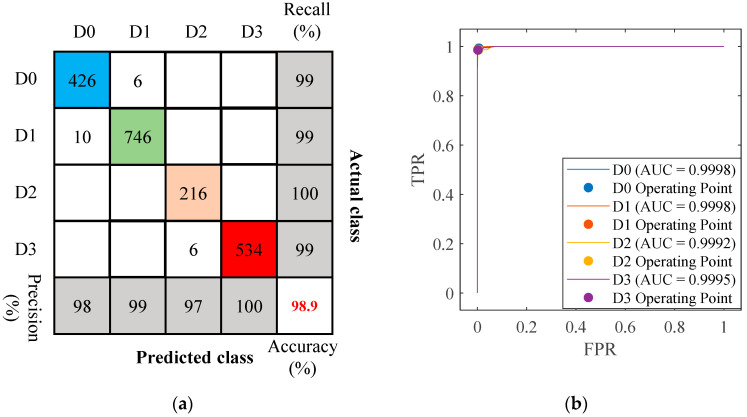
Confusion matrices and ROC curves for wavelet packet spectrum features with dimensions of 256. (**a**) Confusion matrices; (**b**) ROC curves. TPR: True positive rate; FPR: False positive rate.

In the wavelet packet analysis of detection signals, the wavelet packet energy spectrum was divided into 128, 256 and 512 features. When different numbers of wavelet packet energy spectrum features were input into the KNN model, the optimal classification accuracy reached 99.4%, significantly higher than that of models using time-domain and frequency-domain features as input. Among the models with different input feature dimensions, the model using 256-dimensional wavelet packet energy spectrum features achieved high accuracy, with a training time of 14.537 s and a model size of 59 MB. The corresponding confusion matrix and receiver operating characteristic (ROC) curve are shown in Figure 22.

Figure 22b shows the ROC curves and operating points for the four classes. All curves lie close to the top-left corner, and the annotated operating points are near (0, 1), indicating very high recall and extremely low false positive rates across classes. This suggests the model achieves near-perfect separability for each class with good consistency in performance among classes. The accuracies, training times, and model sizes of other models are listed in Table 5.

**Figure 23:**
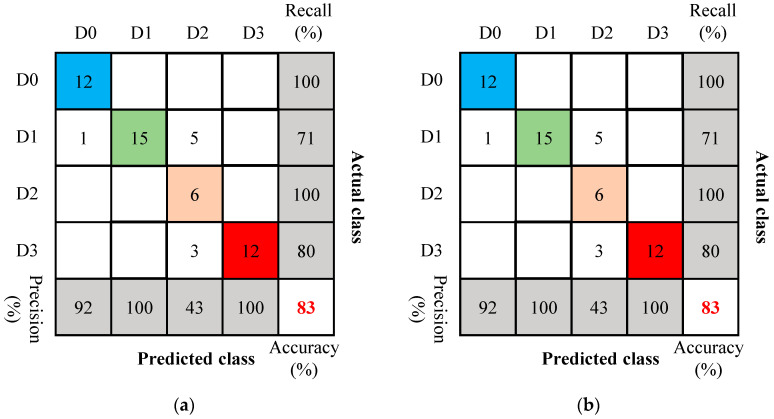
Confusion matrix of single-channel signals. (**a**) G1–S1; (**b**) G2–S2.

To compare the impact of different dataset sizes and excitation-sensing paths on detection accuracy, models were trained using the G1–S1 and G2–S2 datasets under Signal-0. As shown in Section 4.2, each dataset contains 54 samples, with the number of data samples for the four damage states being 12, 21, 6, and 15, respectively. These data were classified using the KNN algorithm, with 256-dimensional wavelet packet energy spectrum features as input, and 10-fold cross-validation was used for model validation. Compared with the model built using all valid data, both models achieved 98.1% accuracy on the validation set, and their confusion matrices are shown in Figure 23. Compared with the 98.9% obtained by the model trained using all valid data reported earlier, this value is slightly lower, mainly because the training data under a single sensing channel are limited in scale and relatively insufficient in feature completeness and richness, which, to some extent, affects the discriminative performance of the model. Nevertheless, under the condition of using only small-scale datasets from a single sensing channel, the established KNN models still exhibited good damage-state classification performance.

#### 5.2.2. Model Generalization Testing

As shown in Section 5.2.1, the model using WPT features as input achieved high accuracy on the training set. However, in practical engineering applications, differences in working conditions, installation positions, and stress wave propagation paths between the test and training dampers may reduce damage detection accuracy.

To evaluate the generalization capability of the trained model, the validation specimen, which is homologous to the primary specimen but lacks rib constraints, was used for testing. The trained model was tested using 54 Signal-0 detection signals from the G1–S1 channel of the validation damper, allowing the evaluation of the model’s applicability and stability on a homologous damper with slight structural differences.

**Figure 24:**
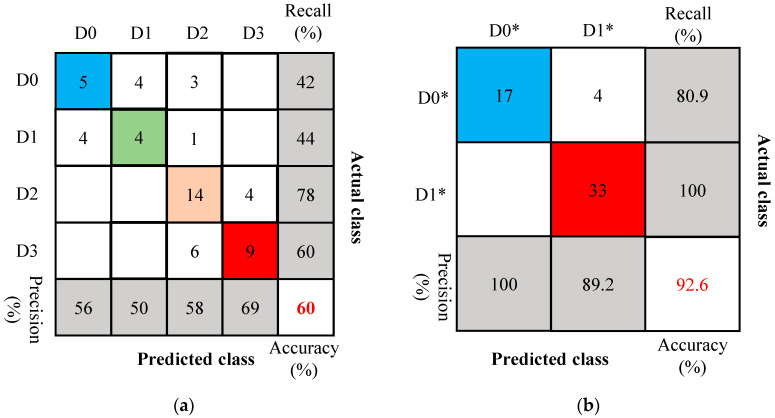
Confusion matrix for 256-dimensional wavelet packet spectrum features in testing. (**a**) The damage degree is divided into four levels; (**b**) The damage degree is divided into two levels.

The confusion matrix of the model using 256-dimensional wavelet packet energy spectrum features as input is shown in Figure 24. This model achieved a test accuracy of 59.3%, which was the highest among the three models, suggesting that the proposed KNN model has some cross-specimen generalization capability for four-class classification, but its performance is still limited. However, in practical engineering applications, the primary concern is usually whether a damper needs to be replaced rather than identifying its exact damage category. Therefore, the original four damage categories were further merged into two classes: healthy and damaged. The healthy state D0* includes the original Healthy (D0) and Damage Progression (D1) states, both of which do not require damper replacement, while the damaged state D1* includes Web Buckling (D2) and Failure (D3), both indicating that the damper should be replaced. After redefining the MSPD damage states into these two classes, the model achieved an accuracy of 92.6% in determining whether damper replacement was required (the accuracies of the other models are listed in Table 6). This result indicates that, although the model shows limited generalization performance in the four-class task, it still has good applicability for practical replacement decision-making.

#### 5.2.3. Comparison with Other Machine Learning Algorithms

By using the 256-dimensional wavelet packet energy spectrum features of all valid data as input, the features were fed into various machine learning classifiers for comparison, including KNN, Decision Tree, Linear Discriminant Analysis (LDA), Support Vector Machine (SVM), kernel-based methods, Naive Bayes, 1D Convolutional Neural Network (1D-CNN), and Deep Neural Network (DNN). The basic characteristics and purposes of these algorithms are summarized in Table 7.

The hyperparameters of all machine learning models were systematically optimized using grid search combined with ten-fold cross-validation. For the KNN model, the number of neighbors *k* was optimized over the range 1–15, and the distance metric was selected from Euclidean, Manhattan, and Minkowski. The final configuration was *k* = 1 with Euclidean distance; for SVM, the kernel type (linear, RBF, polynomial, cubic) and penalty parameter *C* (0.1–100) were adjusted; for Decision Tree, the maximum depth (10–100) and minimum leaf samples (1–10) were optimized; for LDA, the covariance structure (full or diagonal) was selected; for kernel-based methods, the kernel type, regularization parameter Lambda (10^−3^–10^3^), and kernel scale γ were tuned; for Naive Bayes, the core method and kernel function were evaluated. For 1D-CNN and DNN, multiple hyperparameters—including layer numbers, convolution/kernel sizes, fully connected layer sizes, Dropout rates, activation functions, and learning rates—were explored via grid search. Each model’s final hyperparameters were chosen based on the highest mean validation accuracy, ensuring effective feature extraction and robust generalization.

The results show that the KNN model outperforms the others in the task of damper damage detection. This superiority is mainly attributed to its non-parametric nature, insensitivity to small sample sizes, and strong capability to measure distance differences among high-dimensional features such as wavelet packet energy spectra. Therefore, KNN is better able to capture the characteristic differences between various damage states, demonstrating both high accuracy and strong generalization ability. The hyperparameters of other machine learning algorithms and their accuracy on the validation set are shown in Table 8.

## 6. Conclusions

This study verified the feasibility of applying the active sensing method for in situ damage detection of metallic shear plate dampers (MSPDs). Piezoelectric sensors were installed on the damper’s end plates to collect stress wave signals under different damage states, and wavelet packet energy spectrum analysis was employed to extract time–frequency domain features for damage classification using a machine learning model. The proposed approach achieved a maximum classification accuracy of 98.9% for multi-class damage identification and 92.6% for binary classification between healthy and damaged states.

It should be noted that the quasi-static cyclic loading used in this study differs from the high dynamic loading experienced during earthquakes, which may affect material yield stress and stress wave propagation, potentially influencing WPT feature interpretation. Additionally, the long-term performance of the PZT sensor bonding may slightly affect signal amplitude over time, so periodic calibration or alternative attachment methods are recommended to ensure reliable measurements.

The main contributions of this study are as follows:

(1) A novel active-sensing-based damage detection framework for metallic dampers was developed, which enables in situ damage evaluation without interfering with the damper’s normal operation.
(2) A comprehensive experimental program was conducted, combining quasi-static loading tests, mechanical property monitoring, and thermal imaging to define and categorize the damper’s damage states.
(3) The integration of wavelet packet energy features with the KNN algorithm proved effective for classifying different damage levels, demonstrating the potential of combining signal-based features and machine learning in structural health monitoring applications.

In future work, additional experiments will be carried out on dampers with various geometries and sizes to further validate the generalization and robustness of the proposed method. Moreover, approaches such as transfer learning and data augmentation will be explored to adapt the trained model to dampers with varying structural parameters. As more experimental data become available, machine learning models such as Support Vector Machines (SVM), Random Forests (RF), and Deep Neural Networks (DNN) will also be investigated to enhance the method’s generalization capability and automate damage detection.

## Figures and Tables

**Table 1:** Mechanical properties of LY225 and Q355 steel.

Material	Yield Strength (MPa)	Ultimate Strength (MPa)	Elastic Modulus (GPa)
LY225	225	360	206
Q355	345	470	206

**Table 2:** Signal types and their Frequency Band Ranges.

Signal Labeling	Signal-0	Signal-1	Signal-2	Signal-3	Signal-4	Signal-5
Frequency Band Range(kHz)	0–100	0–20	20–40	40–60	60–80	80–100

**Table 3:** Damage states and corresponding displacements of the primary specimen.

Damage States	Healthy	Damage Progression	Web Buckling	Failure
Displacement (mm)	0–4	4–30	30	30–50
Cycle numbers	1–9	10–30	31–36	37–44

**Table 4:** Damage states and corresponding displacements of the validation specimen.

Damage States	Healthy	Damage Progression	Web Buckling	Failure
Displacement (mm)	0–4	4–10	10–30	30–50
Cycle numbers	1–9	10–15	16–28	28–44

**Table 5:** The accuracy, training times, and model sizes of KNN models with different input features.

Input Feature	Accuracy (%)	Training Times (s)	Model Sizes (MB)
Time-domain max amplitudes	53.0	5.933	3
Fre-domain max amplitudes	66.5	4.782	5
The wavelet packet energy spectrum-128	98.8	6.892	30
The wavelet packet energy spectrum-256	98.9	14.537	59
The wavelet packet energy spectrum-512	99.4	61.953	116

**Table 6:** The accuracy of KNN models with different input features for testing.

Input Feature	Accuracy in Four-Class (%)	Accuracy in Two-Class (%)
The wavelet packet energy spectrum-128	53.7	92.6
The wavelet packet energy spectrum-256	59.3	92.6
The wavelet packet energy spectrum-512	38.9	87.0

**Table 7:** Basic characteristics and purposes of the machine learning algorithms used in this study.

Machine-Learning Algorithm	Description
KNN	Non-parametric classifier; classifies samples based on majority vote of nearest neighbors; effective for high-dimensional, small sample datasets.
SVM	Constructs optimal hyperplanes in feature space; supports linear and non-linear classification using kernel functions.
Decision Tree	Tree-structured classifier; splits data based on feature thresholds to maximize class separation; handles non-linear relationships; easy to interpret.
LDA	Statistical method to find a linear combination of features maximizing class separability; assumes normally distributed classes with shared covariance.
Kernel Approximation Classifier	Maps input features to a higher-dimensional space to capture non-linear relationships, including kernel SVMs and other kernel classifiers.
Naïve Bayes	Probabilistic classifier based on Bayes’ theorem; assumes feature independence; computationally efficient for high-dimensional data.
1D-CNN	Deep learning model for sequence or signal data; convolutional layers automatically extract hierarchical temporal or spatial features.
DNN	Multi-layer fully connected network; capable of modeling complex non-linear relationships in high-dimensional data.

**Table 8:** The accuracy of other machine learning algorithms tested on the validation.

Machine-Learning Algorithm	Hyperparameter Setting	Validation Accuracy (%)
SVM	Cubic kernel, automatic kernel scale, penalty parameter = 1, one-vs-one multi-class, data standardization.	91.7
Decision Tree	Gini impurity, max splits = 100, alternative splitting off.	83.8
LDA	Full covariance structure, SVD solver.	75.4
Kernel Approximation Classifier	SVM kernel, automatic dimensionality expansion, automatic Lambda, automatic kernel size, one-vs-one multi-class, data standardization, inference limit = 1000.	83.4
Naïve Bayes	Core Bayesian method, Gaussian kernel, supports unbounded data, data standardization.	63.5
1D-CNN	3 Conv1D layers (Conv1: 5 × 16, Conv2: 3 × 32, Conv3: 3 × 64) with ReLU + BatchNorm; MaxPooling1D after Conv1 & Conv2 (pool size = 2, stride = 2); GlobalAveragePooling1D; Fully connected layer 64 + Dropout 0.3; Output layer softmax; Optimizer: Adam; Initial learning rate = 1 × 10^−3^.	77.2
DNN	512–256–128–64 fully connected layers, BatchNorm, ReLU, Dropout = 0.2, softmax output.	83.5

## Data Availability

The data that support the findings of this study are available from the corresponding author upon reasonable request.

## Abbreviations

The following abbreviations are used in this manuscript:

MSPD	Metallic Shear Plate Dampers
KNN	k-nearest neighbor
SVM	Support Vector Machine
LDA	Linear Discriminant Analysis
DNN	Deep Neural Network
